# Supplementary material for: Serum Phosphorus as a Risk Factor of Metabolic Syndrome in the Elderly in Taiwan: A Large-Population Cohort Study
Source: Nutrients. 2019 Oct 2;11(10):2340. doi: 10.3390/nu11102340 (PMC6835508; doi:10.3390/nu11102340)
Supplement: Supplementary file 1 [file nutrients-11-02340-s001.pdf]

Supplementary Table S1. Numbers of metabolic syndrome, diabetes mellitus and hypertension by age group after follow-up

		Group Age 1-39	*Group Age 40-59*	*Group Age 60-*
Number	Metabolic syndrome (N)	56	79	51
	Diabetes mellitus (N)	7	42	30
	Hypertension (N)	65	121	74
